# Human multipotent stromal cells attenuate lipopolysaccharide-induced acute lung injury in mice via secretion of tumor necrosis factor-α-induced protein 6

**DOI:** 10.1186/scrt68

**Published:** 2011-05-13

**Authors:** Svitlana Danchuk, Joni H Ylostalo, Fokhrul Hossain, Randy Sorge, Austin Ramsey, Ryan W Bonvillain, Joseph A Lasky, Bruce A Bunnell, David A Welsh, Darwin J Prockop, Deborah E Sullivan

**Affiliations:** 1 Department of Medicine, Section of Pulmonary Diseases, Critical Care and Environmental Medicine, Tulane University School of Medicine, 1430 Tulane Avenue, New Orleans, LA 70112, USA; 2 Center for Stem Cell Research and Regenerative Medicine, Tulane University School of Medicine, 1430 Tulane Avenue, New Orleans, LA 70112, USA; 3 Texas A&M Health Science Center, College of Medicine, Institute for Regenerative Medicine at Scott & White, 5701 Airport Road, Module C, Temple, TX 76502, USA; 4 Department of Medicine, Section of Pulmonary/Critical Care Medicine, LSU Health Sciences Center, 1901 Perdido Street, Suite 3205, New Orleans, LA 70112, USA; 5 Department of Microbiology and Immunology, Tulane University School of Medicine, 1430 Tulane Avenue, New Orleans, LA 70112, USA

## Abstract

**Introduction:**

Multipotent stromal cells (MSCs) are currently in clinical trials for a number of inflammatory diseases. Recent studies have demonstrated the ability of MSCs to attenuate inflammation in rodent models of acute lung injury (ALI) suggesting that MSCs may also be beneficial in treating ALI.

**Methods:**

To better understand how human MSCs (hMSCs) may act in ALI, the lungs of immunocompetent mice were exposed to lipopolysaccharide (LPS) and four hours later bone marrow derived hMSCs were delivered by oropharyngeal aspiration (OA). The effect of hMSCs on lung injury was assessed by measuring the lung wet/dry weight ratio and total protein in bronchoalveolar lavage (BAL) fluid 24 or 48 h after LPS. BAL fluid was also analyzed for the presence of inflammatory cells and cytokine expression by multiplex immunoassay. Microarray analysis of total RNA isolated from treated and untreated lungs was performed to elucidate the mechanism(s) involved in hMSC modulation of lung inflammation.

**Results:**

Administration of hMSCs significantly reduced the expression of pro-inflammatory cytokines, neutrophil counts and total protein in bronchoalveolar lavage. There was a concomitant reduction in pulmonary edema. The anti-inflammatory effects of hMSCs were not dependent on localization to the lung, as intraperitoneal administration of hMSCs also attenuated LPS-induced inflammation in the lung. Microarray analysis revealed significant induction of tumor necrosis factor (*TNF*)-α-induced protein 6 (*TNFAIP6/*TSG-6) expression by hMSCs 12 h after OA delivery to LPS-exposed lungs. Knockdown of TSG-6 expression in hMSCs by RNA interference abrogated most of their anti-inflammatory effects. In addition, intra-pulmonary delivery of recombinant human TSG-6 reduced LPS-induced inflammation in the lung.

**Conclusions:**

These results show that hMSCs recapitulate the observed beneficial effects of rodent MSCs in animal models of ALI and suggest that the anti-inflammatory properties of hMSCs in the lung are explained, at least in part, by activation of hMSCs to secrete TSG-6.

## Introduction

Acute lung injury (ALI) and its more severe manifestation, acute respiratory distress syndrome (ARDS), are major complications in critically ill patients. ALI is a syndrome of widespread lung inflammation and increased pulmonary vascular permeability resulting in pulmonary edema, hypoxia and may contribute to multiple organ failure and death. ALI/ARDS is most commonly caused by sepsis, pneumonia, trauma or aspiration of gastric contents. Despite improvements in critical care and mechanical ventilation protocols, the mortality rate for patients with ALI is still 30% to 40% [1-3]. Multipotent stromal cells (MSCs), also known as mesenchymal stem cells, have been proposed as a cellular therapy for ALI.

MSCs are fibroblast-like cells characterized by their ability to self-renew and undergo differentiation into mesenchymal lineage cell types including bone, cartilage, adipose tissue, muscle and tendon [4]. MSCs have been isolated from the bone marrow and the connective tissue of almost all organs including adipose, periosteum, synovial fluid, muscle, hair follicles, root of deciduous teeth, articular cartilage, placenta, dermis, umbilical cord, Wharton's jelly, lung, liver and spleen [4-6]. The interest in MSCs as cellular therapy arises from numerous *in vivo *studies showing that MSCs avoid allorecognition, home to sites of injury, and suppress inflammation as well as immune responses. In addition, MSCs can be rapidly expanded *in vitro *while maintaining their multipotent properties [7]. MSCs are currently in clinical trials for treatment of a number of human diseases including osteogenesis imperfecta, osteoarthritis, graft-versus-host disease, multiple sclerosis, types 1 and 2 diabetes, Crohn's disease, acute kidney injury, acute myocardial infarction, ischemic heart failure, and chronic obstructive pulmonary disease [8].

Intravenous or intra-alveolar administration of MSCs modulates both the inflammatory process and tissue remodeling in experimental models of ALI despite minimal, if any, engraftment (reviewed in [9]). The mechanisms involved are poorly understood; however, increasing data suggest that the protective effects of MSCs are largely mediated through production of paracrine mediators [7,10]. Most of what is known about the therapeutic function of MSCs comes from studies that employed rodent MSCs. It has been suggested that the mechanisms of MSC-meditated immunosuppression may be different between rodent MSCs and human MSCs (hMSCs) in some models [11] but not in others, including the experimental allergic encephalomyelitis model for multiple sclerosis [12,13]. Before proceeding to clinical trials, it is critical to understand the mechanisms integral to the beneficial effects of hMSCs in ALI so that the human physiological response can be accurately predicted.

To elucidate the mechanisms involved in human MSC modulation of inflammation in the lung, we used intra-pulmonary delivery of *E. coli *endotoxin to immunocompetent mice, a well-characterized model of ALI. Here we demonstrated that xenographic transplantation of hMSCs suppress inflammation and lung injury. Taking advantage of the cross-species nature of our experiments, we employed human-specific gene arrays to identify potential anti-inflammatory factors expressed by hMSCs in the injured lung. TNF-α-induced protein 6 (*TNAIP6/*TSG-6) was shown to be highly induced in hMSCS in response to lung injury. Using RNA interference, we demonstrated that the anti-inflammatory properties of hMSCs in the lung are explained, at least in part, by activation of hMSCs to secrete TSG-6.

## Materials and methods

### Cell culture

Adult hMSCs from bone marrow were obtained from the Center for the Preparation and Distribution of Adult Stem Cells [14]. The center has supplied standardized preparations of MSCs enriched for early progenitor cells to over 350 laboratories under the auspices of a National Institutes of Health/National Center for Research Resources (NIH/NCRR) grant (P40 RR17447). Vials of about 0.5 × 10^6 ^cells (passage 1 or 2) were thawed, plated on two-stack culture chambers (Corning, Lowell, MA, USA) in 100 ml/stack of complete culture medium (CCM) at 150 cells/cm^2 ^density, and incubated at 37°C in 5% CO_2 _for six to seven days until 70% confluent. The CCM consisted of α-MEM (Invitrogen, Gaithersburg, MD, USA) containing 17% FBS (lot-selected for rapid growth of hMSCs; Atlanta Biologicals, Lawrenceville, GA, USA); 100 units/ml of penicillin; 100 μg/ml of streptomycin; and 2 mM L-glutamine (Invitrogen). One day before cells were to be administered, medium was changed to antibiotic-free CCM. hMSCs were harvested with 0.25% trypsin/1 mM EDTA (Invitrogen) for 3 minutes at 37°C, washed with PBS (without Ca^2+ ^and Mg^2+^) by centrifugation at 480 × g for 10 minutes at room temperature, resuspended in PBS and administered to mice. IMR-90 human lung fibroblasts (HLFs) were obtained from the American Type Culture Collection (ATCC, Rockville, MD, USA) and cultured according to manufacturer's instructions. HLFs were used at passages 14 to 15 for all experiments.

### Animal model of ALI

Animal care and all animal procedures were approved by the Institutional Animal Care and Use Committee of Tulane University. Eight- to-ten-week-old female BALB/C mice (National Cancer Institute-Frederick, Frederick, MD, USA) were treated with either 1 mg/kg lipopolysaccharide (LPS, Sigma-Aldrich, St. Louis, MO, USA) from *Escherichia coli *(serotype 0111:B4) in 100 μl PBS or an equal volume of PBS, as vehicle control, by oropharyngeal aspiration (OA) as previously described [15]. Briefly, mice were anesthetized with 2% isoflurane vapor (VetOne, Meridian, ID, USA) in oxygen and suspended on a string by the cranial incisors. A droplet was placed in the back of the throat while simultaneously holding the tongue to block the swallow reflex and pinching the nose, forcing the mouse to breathe through its mouth and in the process aspirate the liquid. Four hours after LPS exposure, hMSCs or HLFs (2.5 × 10^5^/100 μl PBS) were given by OA and 30 minutes later a second dose of equal concentration was administered, for a total of 5 × 10^5 ^cells. As the control, 200 μl PBS was delivered as described above. Recombinant human TSG-6 protein (rhTSG-6, R&D Systems, Minneapolis, MN, USA) was administered at 50 μg/200 μl PBS by OA in two equal doses, 30 minutes apart, 1 h and 10 h after LPS delivery to the lung. Mice were anesthetized with 80 mg/kg ketamine plus 8 mg/kg xylazine and euthanized by exsanguination 24 or 48 h after LPS treatment. The right lung was processed for bronchoalveolar lavage (BAL) and the left lung was used for RNA isolation or histology. Dry/wet weight measurements were performed on whole lungs from a separate set of animals.

### Histology

Lungs were perfused with 10% buffered formalin (Sigma-Aldrich) at a pressure of 25 cm H_2_O for 15 to 20 minutes, removed from the animal and placed in fresh 10% neutral buffered formalin for 16 to 20 h at 4°C prior to processing and embedding. Sections (4 μM) from each sample were stained with hematoxylin and eosin (H&E) for histopathological evaluation by two independent experts blinded to the treatment. Whole H&E-stained sections were digitally imaged using an Aperio ScanScope (Aperio Technologies, Vista, CA, USA). TIFF Images of whole lung sections were acquired using the ImageScope program from Aperio. Threshold analysis of whole lung sections, excluding non-parenchymal structures (for example, large blood vessels, airway structural elements, connective structures, and so on), was performed using the NIH ImageJ program. Briefly, RGB color images were split into individual color channels (RGB stack), and analyses were performed using the green channel. The threshold was then adjusted to the highest point that would highlight all areas of the parenchyma while excluding background. The threshold area of total parenchyma was measured and recorded. Then, the threshold was reset to highlight only areas of high pixel density which correlated with lung damage (damage threshold) as determined by microscopic regional image analysis (Supplemental Figure S1 in Additional file 1). Equal threshold values were applied across groups to all images analyzed. Lung injury index was calculated as the ratio of damaged area relative to total parenchyma area and reported as fold increase over control lung (PBS + PBS).

### Bronchoalveolar lavage (BAL) and differential cell counts

Lung lavage was performed immediately after euthanasia with five aliquots consisting of 600 μl of sterile PBS supplemented with 0.4 mM EDTA and protease inhibitor cocktail (Roche, Indianapolis, IN, USA). Aliquots were centrifuged at 1,500 × *g *for five minutes at 4°C to separate cells from supernatant. Supernatant from the first aliquot was stored at -70°C for biochemical analysis and cells from all aliquots were combined and counted using a grid hemocytometer. Differential cell counts were performed after cytocentrifugation and staining with a modified Wright stain (Diff-Quik, Fisher Scientific, Pittsburgh, PA, USA) by counting 300 cells in each of three different viewing fields.

### Lung wet/dry weight ratio

Lungs were dissected immediately after euthanasia and the wet weight was recorded. Lungs were then placed in an incubator at 65°C for 72 h, and the dry weight was determined.

### Cytokine and protein measurements

Measurement of cytokines and chemokines in BAL fluid was performed by multiplex immunoassay using Milliplex mouse cytokine/chemokine 22-plex kit (Millipore, Billerica, MA, USA) following the manufacturer's instructions. MIP-2 and MCP-1 levels were measured with mouse specific Quantikine CXCL2/MIP-2 and CCL2/JE/MCP-1 immunoassays, respectively (R&D Systems). The quantification of mouse IL-6 was performed with mouse IL-6 ELISA MAX™ Deluxe Set from BioLegend (San Diego, CA, USA) according to the manufacturer's instruction. Protein concentration was quantified using the Micro BCA protein assay kit (Pierce, Rockford, IL, USA).

### RNA isolation and RT-PCR

Total RNA was obtained from lung tissue homogenized in TriPure Isolation Reagent (Roche) and was purified with the RNeasy mini kit (Qiagen, Valencia, CA, USA). Total RNA concentration was measured using NanoDrop spectrophotometer (Thermo Scientific Nanodrop, Nanodrop Technologies, Wilmington, DE, USA) and quality assessed by the 260/280 and 230/260 ratios. Reverse transcription was carried out with 1 μg of total RNA using a First Strand cDNA Synthesis kit (Bio-Rad, Hercules, CA, USA) following the instructions provided by the manufacturer. Quantitative real-time PCR (qRT-PCR) was performed using a 40-cycle two-step PCR (95°C for 15 sec followed by 60°C for 1 minute) with sequence-specific primer pairs using the iCycler IQ real-time detection System (Bio-Rad). qRT-PCR for human TSG-6 and GAPDH mRNA was performed using human-specific TSG-6 primers/probe [15] or human GAPDH Taq-Man Gene Expression Assay (Hs00266705_g1, Applied Biosystems, Foster City, CA, USA) and Taqman Universal PCR Master Mix (Applied Biosystems) with 200 ng of cDNA. The number of viable hMSCs in mouse lung at the time of euthanasia was evaluated as a percentage of administered hMSCs based on qRT-PCR results for human GAPDH mRNA as previously described [16]. Human keratinocyte growth factor (KGF) and interleukin 1 receptor antagonist (IL1RN) mRNA levels were assayed using Taq-Man Gene Expression Assays Hs00384281_m1 and Hs00893625_m1, respectively (Applied Biosystems). The species specificity of each primer set was confirmed by analyzing RNA isolated from mouse lung and hMSCs in each reaction.

### Microarray analysis

RNA was isolated from whole lungs of LPS- (LPS + hMSC) or PBS-exposed (PBS + hMSC) mice 12 h after OA administration of 5 × 10^5 ^hMSCs. Control RNA was isolated from LPS-exposed lung treated with PBS (LPS + PBS). To establish baseline levels of gene expression in hMSCs prior to delivery to the lung, 5 × 10^5 ^hMSCs were added to LPS- (LPS + hMSCs *in vitro *mix) or PBS- (PBS + hMSCs *in vitro *mix) exposed mouse lung immediately prior to homogenization. RNA extracts from samples containing hMSCs were analyzed for human GAPDH mRNA by qRT-PCR to estimate the amount of human RNA in each sample. To allow comparisons between samples containing both human and mouse RNA, RNA extracts were diluted to contain equal levels of human RNA. The adjusted RNA samples (approximately 3 μg of total RNA) were assayed on a human (HG-U133 Plus 2.0) microarray (Affymetrix, Santa Clara, CA, USA). An aliquot of total RNA extracted from LPS-exposed lung treated with PBS, equal in concentration to the adjusted RNA sample from LPS-exposed lung treated with hMSCs, was also included to detect cross-hybridization of mouse RNA to the human chip. The data were analyzed using Partek Genomics Suite 6.5 (Partek Inc., St. Louis, MO, USA) and filtered for cross-hybridization (CV >0.5 and call >33%) as previously described [16]. Values were expressed as fold changes relative to the signal intensities of controls. For a human gene to be considered significantly up-regulated in the LPS + hMSC sample the expression in the human array had to be at least two-fold higher than in the 1) LPS + PBS sample, 2) PBS + hMSC sample, 3) LPS + hMSC *in vitro *mix sample, and 4) PBS + hMSC *in vitro *mix sample.

### siRNA transfection

Target hMSCs for transfection were cultured in antibiotic-free CCM and harvested as described above. About 1 × 10^6 ^hMSCs were resuspended in 100 μl resuspension buffer R (Neon Transfection System, Invitrogen, Carlsbad, CA, USA) with 1 μM siRNA for TSG-6 (sc-39819; Santa Cruz Biotechnology, Santa Cruz, CA, USA) or control non-silencing siRNA (sc-37007; Santa Cruz Biotechnology) and transfected in 100 μl Neon tip with Neon transfection system (Invitrogen) using two pulses (1,400 V input pulse voltage/20 ms input pulse width). Transfected hMSCs were plated on 10 cm^2 ^tissue culture dishes in 7.5 ml antibiotic-free CCM for 24 h.

### Myeloperoxidase (MPO) activity

MPO activity in BAL fluid was measured by continually monitoring the change in absorbance of MPO reactions at 460 nm for a period of five minutes using the Synergy HT multi-detection microplate reader (Bio-Tek Instruments, Winooski, VT, USA). Assays were conducted at 25°C in a final volume of 100 μl. The reaction buffer contained 50 mM potassium phosphate (pH 6.0), 0.0005% (volume/volume) H_2_O_2 _and 0.167 mg/ml o-dianisidine dihydrochloride (Sigma-Aldrich, St. Louis, MO, USA). MPO activity in BAL fluid was expressed in units/min/ml using a standard curve with 0 to 0.625 units/ml human leukocyte myeloperoxidase (Sigma-Aldrich).

### Statistical analyses

Data are shown as mean ± SEM. Comparisons between the two groups were made using unpaired, two-tailed Student's *t *tests. *P *< 0.05 was considered statistically significant. To assess the likely contribution of Type 1 error due to multiple analyses performed in the multiplex immunoassays, we calculated *P-*values for the null hypothesis based on the exact confidence intervals for samples from the binomial distribution [17,18].

## Results

To elucidate the role of hMSCs in ALI, the lungs of immunocompetent mice were exposed to LPS and 4 h later 5 × 10^5 ^hMSCs, or HLFs as control, were delivered by OA. At 24 and 48 h after LPS, animals were euthanized by exanguination and lungs processed either for BAL, RNA extraction, histology or lung wet/dry weight measurements to assess inflammation and lung injury. All experiments were repeated at least two times with six animals per group, except where indicated.

### Effect of hMSCs on LPS-induced pulmonary edema and microvascular permeability

**Figure 1:**
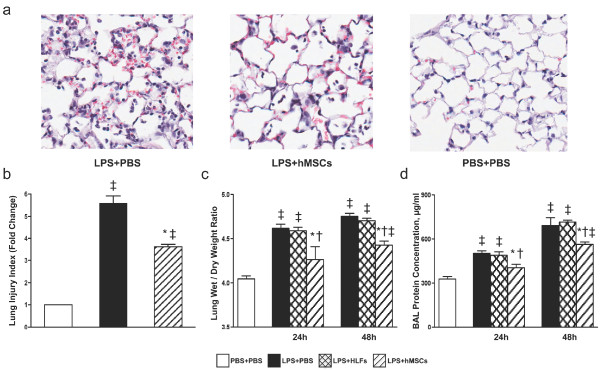
**hMSCs decrease LPS-induced lung injury in immunocompetent mice**. BALB/C mice were treated with LPS or PBS via oropharyngeal aspiration (OA) and four hours later 5 × 10^5 ^hMSCs and HLFs or PBS as controls, were delivered by OA. Lung injury was assessed 24 and 48 h later. (**a**) Representative images of hematoxylin and eosin staining of lung sections demonstrate reduced exudation and cellularity in hMSC treated lungs 48 h after LPS. (**b**) A lung injury index was quantified by threshold image analysis of whole lung sections and reported as fold change over control (PBS + PBS). (**c**) Pulmonary edema as measured by lung wet/dry weight ratio was significantly reduced in hMSC treated groups (*n = *7 to 8 per group). (**d**) Total protein in BALF was also reduced in the hMSC treated groups (*n = *4 to 6 per group). Data are presented as mean ± SEM. *P < 0.05 compared with LPS + PBS treated mice; † *P *< 0.05 compared with LPS + HLF treated group, and ‡ *P *< 0.0007 versus mice treated with PBS + PBS.

Histological examination of LPS-exposed lungs at 48 h showed widespread septal thickening and interstitial neutrophil infiltration, as well as significant air-space cellularity and exudation; all of which were decreased in lungs treated with hMSCs (Figure 1a). The alveolitis induced by LPS is patchy; therefore, we employed threshold analysis of H&E stained lung sections to quantify lung injury (Supplemental Figure S1 in Additional file 1). Lungs from mice exposed to LPS had a lung injury index of 5.6, whereas lungs from mice treated with hMSCs after LPS-exposure had a significantly lower lung injury index of 3.6 (*P *< 0.05) (Figure 1b).

Pulmonary edema and total protein in BAL fluid were assessed 24 h and 48 h after LPS as measures of lung injury and vascular leak. LPS-exposed mice displayed substantial pulmonary edema as indicated by an increased lung wet/dry weight ratio (*P *< 0.0007). Treatment with hMSCs significantly decreased the pulmonary edema at both 24 and 48 h (*P *< 0.05) (Figure 1c). A similar pattern was seen in the total protein concentrations in BAL fluid (Figure 1d) consistent with a decrease in microvascular permeability. Administration of control HLFs had no effect on lung wet/dry weight ratio or BAL fluid protein concentration (Figure 1c, d).

### Effects of hMSCs on inflammation

**Figure 2:**
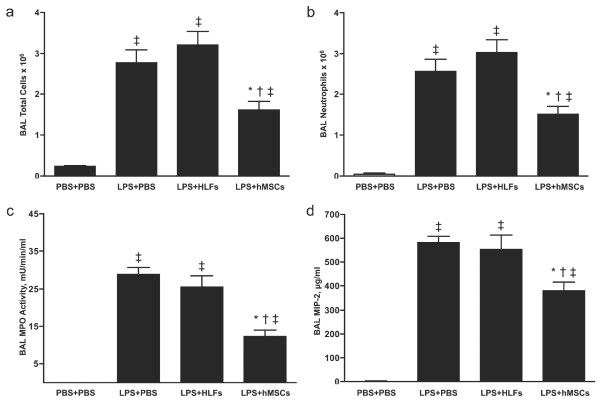
**hMSCs attenuate LPS-induced lung inflammation in immunocompetent mice**. BALB/C mice were treated with LPS or PBS via oropharyngeal aspiration (OA) and four hours later 5 × 10^5 ^hMSCs and HLFs or PBS as controls, were delivered by OA. Differential cell counts, myeloperoxidase (MPO) enzyme activity assay and ELISA for MIP-2 were performed on BAL fluid samples collected 24 h after LPS exposure. Total cells (**a**), neutrophils (**b**), myeloperoxidase (MPO) activity (**c**) and MIP-2 levels (**d**) were all significantly reduced in LPS-exposed lungs after treatment with hMSCs. HLFs had no effect on any of these measures. Data are expressed as mean ± SEM (n *= *6 per group). *P < 0.05 compared with LPS + PBS treated mice; † *P *< 0.05 compared with LPS + HLF treated mice, and ‡ *P *< 0.01 versus mice treated with PBS + PBS.

The total cell number in the BAL fluid was increased approximately 10-fold 24 h after LPS exposure (Figure 2a). This increase was largely due to infiltration of neutrophils, which comprised more than 90% of cells in the BAL fluid (Figure 2b). Administration of hMSCs significantly reduced the total number of neutrophils in the BAL fluid from approximately 2.6 × 10^6 ^to 1.5 × 10^6^. In contrast, delivery of an equal number of HLFs resulted in a slight increase in total cells and neutrophils in BAL fluid. Consistent with these findings, MPO, an enzyme marker of neutrophilic infiltration, was significantly lower (*P *< 0.0001) in BAL fluid from mice treated with hMSCs and unchanged in BAL fluid from mice treated with HLFs (Figure 2c). Since neutrophil counts in BAL fluid were not significantly different from total cell counts, we used total cell counts for subsequent analyses.

**Figure 3:**
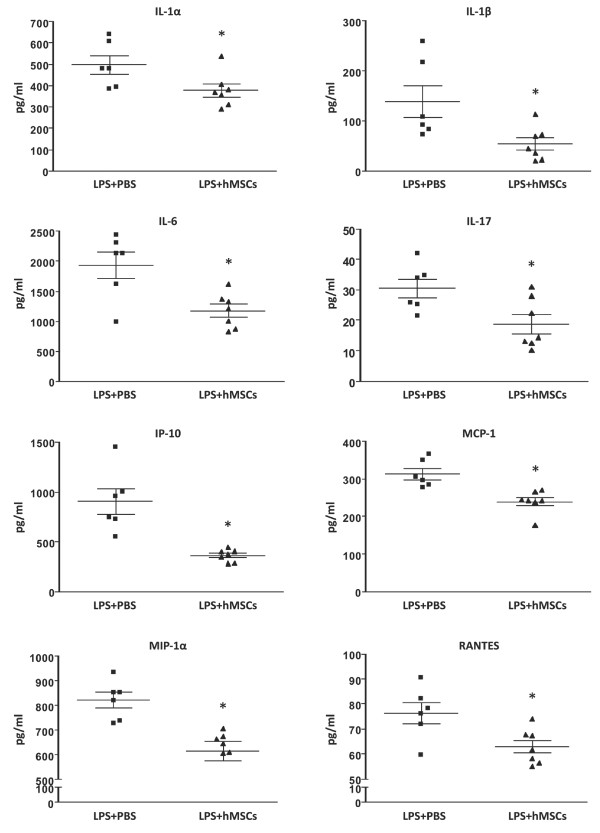
**Proinflammatory cytokine and chemokine expression is decreased by hMSC administration**. Twenty-four hours after LPS exposure, IL-1β, IL-6, IL-17, IP-10, MCP-1, MIP-1α and RANTES levels were measured by multiplex immunoassay in BAL fluid. Data are expressed as mean ± SEM (n *= *6 per group). *P < 0.05

Macrophage inflammatory protein (MIP-2) is a potent chemoattractant for neutrophils. A previous study showed that MIP-2 was increased after exposure of lungs to LPS and mouse MSCs (mMSCs) decreased its expression [19]. To determine if hMSCs decrease expression of MIP-2, BAL fluid was analyzed by ELISA. MIP-2 was undetectable in PBS-treated control mice and increased to approximately 600 mg/ml in response to LPS. Treatment with hMSCs, but not HLFs, resulted in a significant decrease (*P *< 0.05) in MIP-2 levels (Figure 2d). To further characterize the effect of hMSCs on the inflammatory response to LPS, the BAL fluid was assayed for 22 additional cytokines/chemokines using a Milliplex mouse cytokine/chemokine panel. Nineteen of the 22 cytokines/chemokines were detectable in LPS-exposed lungs and 12 were decreased by OA hMSCs (Supplemental Table S1 in Additional file 2). Treatment with hMSCs significantly decreased (*P *< 0.05) expression of the proinflammatory cytokines IL-1α, IL-1β, IL-6, IL-17 and the proinflammatory chemokines IP-10, MCP-1, MIP-1α and RANTES (Figure 3). G-CSF, a cytokine that recruits and activates neutrophils, was also significantly reduced by hMSC administration. TNF-α levels were not affected by hMSC administration. Levels of IL-10, an anti-inflammatory cytokine, were very low in the lungs of LPS-exposed mice and were slightly increased in response to hMSC administration. The calculated probability that the significant results we observed are due only to Type I error is *P *< 0.0001. Moreover, independent ELISAs for IL-6 and MCP-1 confirmed the accuracy of the multiplex immunoassay and further showed that HLFs had no effect on the levels of these cytokines (Supplemental Figure S2 in Additional file 3).

**Figure 4:**
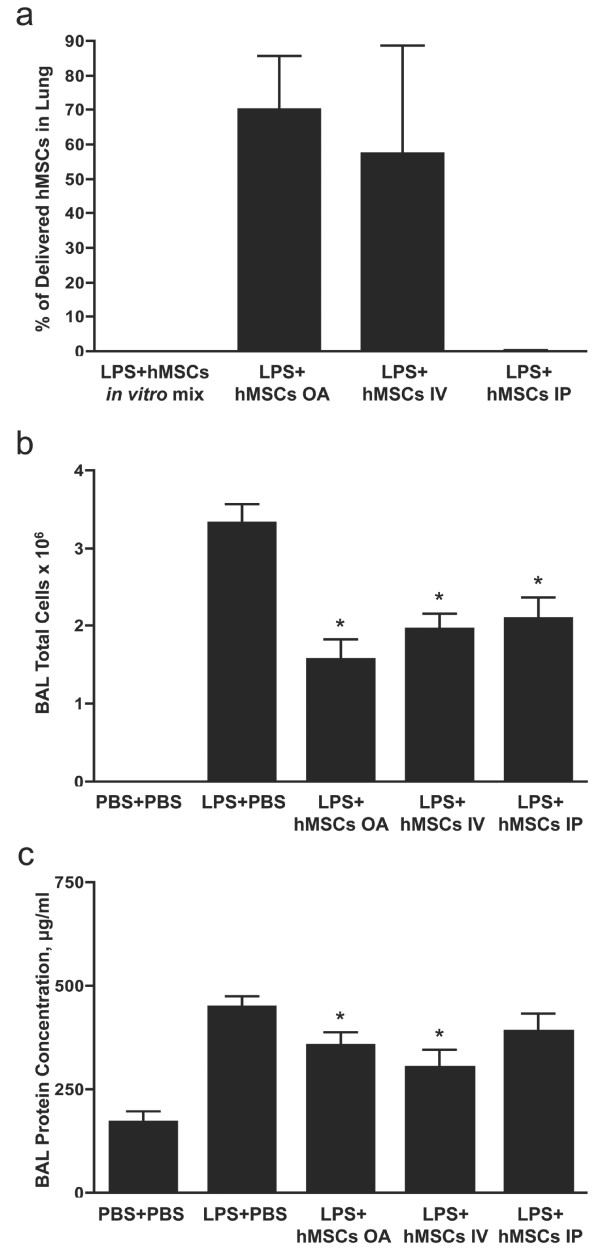
**hMSCs suppress lung inflammation regardless of route of administration**. A total of 5 × 10^5 ^hMSCs were administered OA, IV or IP four hours after LPS exposure. (**a**) The percentage of delivered hMSCs in the lung after one hour was measured by qRT-PCR of human GAPDH mRNA. (**b**) Total cell counts in BAL fluid were significantly decreased by hMSC administration via all three routes. (**c**) Total protein in BAL fluid was slightly decreased by IP delivered MSCs and significantly decreased by both OA and IV delivered MSCs. Data are expressed as mean ± SEM (n *= *5 to 6 per group), *P < 0.05 compared with LPS + PBS.

To determine if the anti-inflammatory function of hMSCs required direct contact with lung epithelium, 5 × 10^5 ^hMSCs were delivered intravenously via the jugular vein (IV), intraperitoneally (IP), or by OA four hours after LPS exposure. IV and OA administration resulted in approximately equal numbers of hMSCs in the lung one hour after delivery as evaluated by real-time RT-PCR assays for live human cells (Figure 4a). In contrast, no hMSCs could be detected in the lung after IP administration. Twenty-four hours after LPS exposure, animals were euthanized and BAL carried out. Total cells in BAL fluid were measured as an index of lung inflammation. Surprisingly, hMSCs significantly suppressed inflammatory cell accumulation in the lung regardless of the route of administration (Figure 4b). Total protein in BAL fluid was significantly decreased by hMSCs delivered via OA or IV (Figure 4c). Although there was a trend toward lower protein concentrations in BAL fluid from animals receiving hMSCs IP, the differences were not statistically significant. These findings support the hypothesis that the anti-inflammatory properties of hMSCs are mediated by paracrine factors.

### hMSC upregulate expression of anti-inflammatory genes in response to inflammation

To elucidate the mechanism(s) involved in hMSC modulation of lung inflammation, we performed microarray analysis taking advantage of the cross-species nature of our experiments. Human MSCs (5 × 10^5^) were delivered by OA to the lungs of mice four hours after treatment with LPS or PBS. Total RNA was isolated from lungs 12 h later, a time at which assays for human GAPDH mRNA indicated there were adequate amounts of human mRNA for assays. To account for any differences in the number of human cells among samples, the amount of RNA analyzed from each sample was adjusted to provide equivalent amounts of human GAPDH mRNA as previously described [14]. The adjusted RNA samples were analyzed on a human microarray. After filtering for cross-hybridization with mouse mRNA isolated from LPS + PBS lung, the data indicated that 53 human genes were significantly upregulated in the LPS-exposed lung compared to LPS + hMSCs *in vitro *mix (Table 1, and Supplemental Figure S3 in Additional file 4). Microarray data have been deposited in Gene Expression Omnibus (accession number [GEO:GSE26567]).

**Table 1:** The top 10 transcripts of hMSCs, up-regulated in lung after OA delivery to LPS exposed mice

Gene name	Gene symbol	LPS + hMSCs vs LPS + hMSCs *in vitro *mix	LPS + hMSCs vs PBS + hMSCs	LPS + hMSCs vs LPS + PBS
Interferon-induced protein with tetratricopeptide repeats 3	*IFIT3*	29	29	31
**Tumor necrosis factor, alpha-induced protein 6**	***TNFAIP6***	**25**	**3**	**24**
Interleukin 8	*IL8*	19	10	23
Chemokine (C-X-C motif) ligand 6	*CXCL6*	18	18	17
Superoxide dismutase 2, mitochondrial	*SOD2*	16	12	19
Chemokine (C-X-C motif) ligand 5	*CXCL5*	11	9	8
Spermidine/spermine N1-acetyltransferase 1	*SAT1*	10	7	14
Integrin-binding sialoprotein	*IBSP*	7	6	6
Pregnancy-associated plasma protein A, pappalysin 1	*PAPPA*	6	8	6
ATP-binding cassette, sub-family A, member 1	*ABCA1*	6	3	5

Shown are fold changes in mRNA levels assayed by microarray (Supplemental Figure S1 in Additional file 1). LPS + hMSCs is RNA extracted from LPS-exposed lung 12 h after treatment with hMSCs; LPS + hMSCs *in vitro *mix is hMSCs added to lung tissue from LPS-exposed mouse immediately prior to RNA isolation; PBS + hMSCs is RNA extracted from PBS-exposed lung 12 h after treatment with hMSCs; LPS + PBS is RNA extracted from LPS-exposed lung 12 h after treatment with PBS. hMSCs, human multipotent stromal cells; LPS, lipopolysaccharide; OA, oropharyngeal aspiration; PBS, phosphate buffered saline.

**Figure 5:**
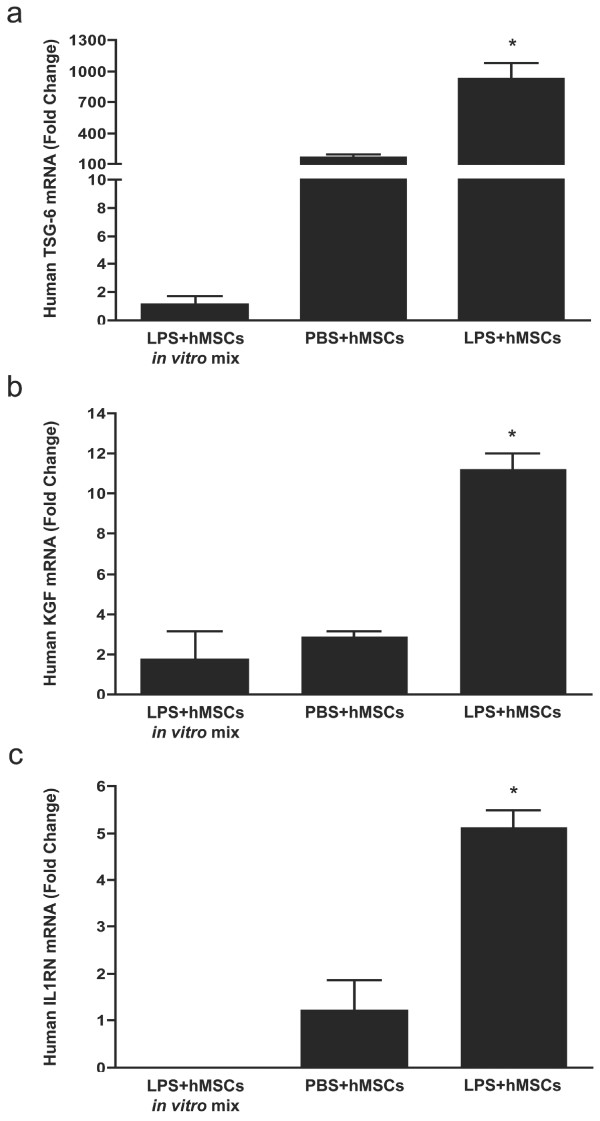
**hMSCs upregulate expression of anti-inflammatory genes in response to LPS-treated lung**. Twelve hours after delivery of 5 × 10^5 ^hMSCs to LPS- or PBS-exposed lungs, total RNA was isolated from lungs and analyzed by quantitative RT-PCR for mRNA expression of (**a**) human *TNFAIP6*, (**b**) human *KGF*, or (**c**) human *IL1RN*. Values represent fold increase over values obtained for LPS + hMSCs *in vitro *mix. Data are shown as the mean ± SEM (n *= *3 per group). * *P *< 0.01 versus PBS + hMSCs; † *P *< 0.05 compared with PBS + PBS; and ‡ *P *< 0.05 compared with LPS + PBS.

One of the most highly upregulated human genes identified in LPS-exposed lung encodes TNF-α-induced protein 6 (TSG-6). TSG-6 is a potent anti-inflammatory factor that was previously shown to play a role in intravenous hMSC improvement of myocardial infarction [20,21]. Quantitative real-time RT-PCR (qRT-PCR) analysis of the same RNA samples used for microarray analysis demonstrated that hMSCs delivered to a PBS-exposed lung upregulated their expression of TSG-6 mRNA approximately 100-fold over that in hMSCs prior to delivery to the lung (PBS + hMSCs *in vitro *mix). hMSCs delivered to LPS exposed lungs had even higher levels (approximately 900-fold) of TSG-6 mRNA expression (Figure 5a).

MSC expression of keratinocyte growth factor (*KGF*) was previously shown to be essential for the therapeutic effects of hMSCs in an *ex vivo *perfused lung model of ALI [22]. No change in expression of *KGF *was detected by the microarray analysis performed here. However, qRT-PCR analysis of the RNA samples used for the microarrays demonstrated an approximately 11-fold induction in *KGF *expression in LPS + hMSCs exposed lung. hMSCs delivered to a PBS-exposed lung did not upregulate expression of *KGF *(Figure 5b).

Interleukin 1 receptor antagonist (IL-1RN), has been implicated in the anti-inflammatory function of MSCs [23]. IL-1RN mRNA levels were not detectable by qRT-PCR in hMSCs prior to delivery to the lung (PBS + hMSCs *in vitro *mix). However, hMSCs in the PBS-exposed lungs expressed low levels of IL-1RN mRNA (C_T_'s of 35 to 37) and hMSCs in LPS-exposed lungs expressed approximately five-fold higher levels of IL-1RN mRNA (Figure 5c).

### Effect of TSG-6 on lung inflammation

**Figure 6:**
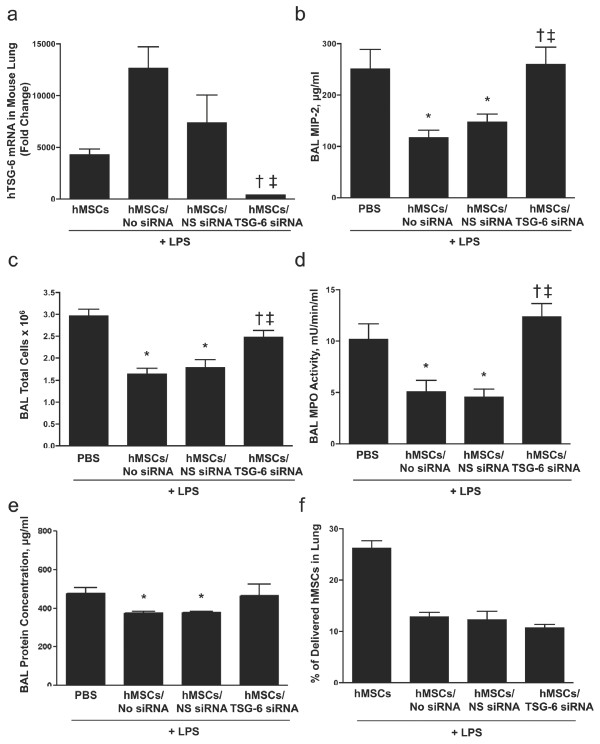
**Knockdown of TSG-6 mRNA in hMSCs reduces their anti-inflammatory effects**. Mice were treated with LPS and four hours later 5 × 10^5 ^hMSCs that were mock transfected (hMSCs/No siRNA), transfected with non-silencing siRNA (hMSCs/NS siRNA), or transfected with TSG-6 siRNA (hMSCs/TSG-6 siRNA) were delivered by OA. Human TSG-6 mRNA levels in the lung, as well as markers of inflammation and injury in BAL fluid were assessed 24 hours later. (**a**) qRT-PCR analyses demonstrated significantly reduced levels of human TSG-6 mRNA in lungs of mice treated with hMSCs/TSG-6 siRNA compared to those treated with hMSCs/No siRNA or hMSCs/NS siRNA. (**b**) MIP-2 concentrations (**c**) total cell counts, and (**d**) MPO activity and (**e**) total protein concentration in the BAL fluid was attenuated in animals treated with hMSCs/No siRNA or hMSCs/NS siRNA but not hMSCs/TSG-6 siRNA. (**f**) The percentage of delivered hMSCs in the lung at the time of human TSG-6 mRNA analysis was determined by measuring human GAPDH mRNA levels and calculating the number of cells from a standard curve as previously described [16]. Data are presented as means ± SEM (n = 6 per group). *P < 0.05 compared with LPS + PBS exposed mice; † *P *< 0.05 compared with mice treated with hMSCs/No siRNA, and ‡ *P *< 0.05 versus mice treated with hMSCs/NS siRNA.

To determine if TSG-6 mediates the therapeutic benefits of hMSCs, cells were transiently transfected with TSG-6 siRNA by electroporation. TSG-6 siRNA significantly knocked down basal levels of TSG-6 mRNA (Supplemental Figure S4 in Additional file 5) in hMSCs. Mock transfection (No siRNA) or transfection with a non-silencing (NS) siRNA resulted in slightly higher expression of TSG-6 mRNA compared to hMSCs that were not transfected (Supplemental Figure S4 in Additional file 5), suggesting that the electroporation procedure itself induces expression of *TNFAIP6 *in hMSCs. Equal amounts (5 × 10^5^) of hMSCs transfected with TSG-6 siRNA, NS siRNA or mock transfected were administered to mice four hours after LPS exposure. Lungs were either lavaged or processed for RNA extraction 24 h after LPS exposure. qRT-PCR analysis showed that lungs receiving either untransfected, mock transfected or NS siRNA transfected hMSCs had significantly higher levels of human TSG-6 mRNA compared to lungs that received hMSCs transfected with TSG-6 siRNA (Figure 6a). Importantly, LPS induced increases in MIP-2 expression (Figure 6b), total cell number (Figure 6c), MPO activity (Figure 6d) and total protein (Figure 6e) in BAL fluid were all significantly decreased by delivery of mock transfected or NS-siRNA transfected hMSCs, but not TSG-6 siRNA transfected hMSCs despite similar numbers of the different transfected hMSCs in the lung 24 h after LPS exposure (Figure 6f).

**Figure 7:**
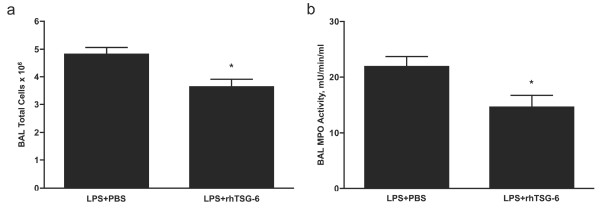
**Recombinant human TSG-6 protein (rhTSG-6) attenuates LPS induced lung inflammation**. A total of 25 μg of rhTSG-6 protein was administered by OA 1 h and again 10 h after LPS-induced lung injury. Total cell counts (**a**) and MPO activity (**b**) in BAL fluid are significantly lower in rhTSG-6 treated mouse lung. Data are shown as mean ± SEM (n *= *5, LPS + PBS or n *= *8 LPS + rhTSG-6). * *P *< 0.05 compared with LPS + PBS.

Analysis of BAL fluid from these mice using a Milliplex mouse cytokine/chemokine panel further revealed that TSG-6 mRNA knockdown abrogated most, but not all, of the anti-inflammatory effects of hMSCs in the LPS-exposed lung (Supplemental Table S2 in Additional file 6). For example, TSG-6 siRNA had no effect on hMSC-mediated suppression of IL-1α, IL-1β IL-7 and IL-13. Consistent with these results, delivery of recombinant human TSG-6 significantly decreased total cell numbers (Figure 7a), and MPO activity (Figure 7b) in the BAL fluid of LPS-exposed mice. These results suggest that TSG-6 mediates, in part, the immunosuppressive function of hMSCs.

## Discussion

Human MSCs attenuated LPS-induced lung inflammation and injury in immunocompetent mice. In response to the pro-inflammatory environment in the LPS exposed lung, hMSCs upregulated expression of a number of genes. Some of these genes encode secreted factors with known anti-inflammatory properties. One of the most highly upregulated genes was *TNFAIP6 *that encodes TSG-6. Knockdown of TSG-6 mRNA expression in hMSCs abrogated most of their anti-inflammatory effects. In addition, intra-pulmonary delivery of rhTSG-6 reduced LPS-induced inflammation in the lung. These results suggest that hMSCs in the lung upregulated expression of *TNFAIP6 *and the secreted TSG-6 decreased neutrophil accumulation and lung injury.

Previous studies have demonstrated the ability of murine MSCs to attenuate inflammation and lung injury in rodent models of ALI [19,24,25]. Recently, the ability of human MSCs to restore alveolar epithelial fluid transport and lung fluid balance was demonstrated in a study employing an *ex vivo *perfused human lung preparation injured by *E. coli *endotoxin [22]. These studies have ignited much interest in hMSCs as cellular therapy for ALI. However, little is known about the mechanisms by which MSCs modulate lung inflammation and repair. The anti-inflammatory effects of mouse MSCs in the lung have been associated with MSC secretion of interleukin 1 receptor antagonist (IL1RN) [23] and TGF-β_1 _[26]. It is not clear how closely mMSCs resemble hMSCs. Certainly these two populations of cells share many features; however, there is some evidence that mMSCs and hMSCs differ in their response to inflammatory cytokines. For example, Ren *et al. *showed *in vitro *that inflammatory cytokines induce the expression of indoleamine 2,3-dioxygenase (*IDO*), but not inducible nitric oxide synthase (*iNOS*), in human MSCs, whereas in mouse MSCs the opposite is true [11]. The goal of our study was to investigate the mechanism(s) by which human MSCs modulate inflammation in the lung.

For these studies we delivered human MSCs to immunocompetent mice after intrapulmonary delivery of LPS, a well-characterized mouse model of direct lung injury that recapitulates many of the features of human ALI including patchy intra-alveolar neutrophil infiltrates and changes in epithelial permeability. A similar cross-species strategy was used to elucidate the mechanism(s) by which hMSCs improve heart function after myocardial infarction [16]. In that study, hMSCs were delivered to immunodeficient mice where they provided numerous endogenous markers for the cells and caused no obvious cross-species artifacts.

Our results demonstrated that hMSCs improved lung histology and reduced pulmonary edema in immunocompetent mice (Figure 1) similar to what has been reported for syngeneic mMSCs in this model [19,24]. The improvement in lung injury was associated with a decrease in proinflammatory cytokines and chemokines, including some (IL-1α, IL-1β, MIP-1α and MIP-2) that function as chemoattractants and activators of neutrophils (Figures 2d and 3). Consistent with these results, hMSC treated lungs had significantly fewer neutrophils, the major mediator of lung injury in this model (Figure 2b). TNF-α levels in BAL fluid were not changed with hMSC treatment and although there was a trend toward increased IL-10 in BAL fluid the levels of IL-10 were very low (Supplemental Table S1 in Additional file 2). Administration of HLFs had no beneficial effects (Figure 2 and Supplemental Figure S2 in Additional file 3). These results suggest that hMSCs protected the lung primarily by preventing recruitment and/or activation of neutrophils. In a study similar to ours, mouse MSCs given four hours after LPS attenuated lung injury and reduced the levels of TNF-α and MIP-2 in BAL fluid. However, neutrophil influx or activation was not ameliorated [19]. The differences in the MSC effects on neutrophil influx may be explained by intrinsic differences in MSCs from humans versus mice, but could also be explained by differences in experimental conditions between the studies, including strain of mice used, dose of LPS and method of intra-pulmonary delivery.

The ability of human MSCs to attenuate lung inflammation and injury in an immunocompetent mouse suggests that the anti-inflammatory action of hMSCs in the lung is non-major histocompatibility complex (MHC)-restricted and further, that hMSCs are to some degree immune privileged due to their immune modulatory effects, as others have suggested [6]. These results support the use of allogeneic hMSCs in patients.

Our finding that hMSCs attenuated lung inflammation when administered intravenously (Figure 4) was not surprising since most of the injected cells are initially trapped in the lung [16]. However, this is the first report that MSCs delivered intraperitoneally attenuate inflammation in the lung (Figure 4b, c). The hMSCs did not travel to the lung after IP injection as indicated by the absence of human GAPDH mRNA in the lung one hour (Figure 4a) or six hours (data not shown) after injection. The fate of the cells is currently under investigation. The IP route of delivery was not as effective as OA and IV routes in reducing LPS-induced lung injury. For these experiments, we used a dose of hMSCs (5 × 10^5^) that we determined to be optimal for OA delivery. It is possible that increasing the number of hMSCs administered IP would provide better protection. Alternatively, our results may suggest that the factor or factors responsible for hMSC suppression of inflammation can function systemically, whereas factors that enhance lung repair may work best when delivered locally. Regardless, these results strongly suggest that the beneficial properties of hMSCs are predominantly mediated through secretion of factors that act in paracrine fashion.

The upregulation of *TNFAIP6 *by hMSCs in response to the inflammatory milieu of the LPS-exposed lung (Table 1) was of special interest because TSG-6 has previously been shown to play a role in the beneficial effects of hMSCs [16]. TSG-6 is a 35 kDa, secreted protein produced by many cell types in response to TNF-α and IL-1β [20,21]. hMSCs in the LPS-exposed lung upregulated their TSG-6 mRNA expression more than 900-fold (Figure 5a). TSG-6 is composed mainly of contiguous Link and CUB modules that interact with a broad spectrum of glycosaminoglycans and proteins, including HA, chondroitin 4-sulphate, dermatan sulfate, heparan sulfate, heparin, inter-α-inhibitor, versican, aggrecan, thrombospondin-1, and PTX3 [20]. The anti-inflammatory properties of TSG-6 are well documented and the mechanisms are beginning to be elucidated. TSG-6 inhibits the inflammatory network of proteases by increasing the inhibitory activity of inter-α-inhibitor and bikunin [27]. TSG-6 also specifically binds and sequesters hyaluronan fragments and has been shown to be a potent inhibitor of neutrophil activation and migration, as well as tissue remodeling [28], through up-regulation of Cox-2 and prostaglandin D2 expression [28,29]. The precise mechanism(s) by which TSG-6 protects the lung from acute inflammation remains to be determined.

Knockdown of TSG-6 mRNA expression in hMSCs did not completely abrogate their anti-inflammatory effects (Figure 6) suggesting that additional factors expressed by hMSCs play a role in protecting the lung from LPS-induced inflammation and injury. The hMSCs used in our studies expressed high levels of KGF in the lung (Figure 5b). Lee *et al. *showed in an isolated, perfused human lung model challenged with endotoxin, that secretion of KGF was essential for the beneficial effect of hMSCs on alveolar epithelial fluid transport [22]. In another study, Aguilar *et al. *[30] showed that MSCs transfected with a tetracycline-inducible *KGF *construct partially protected mice from bleomycin-induced pulmonary fibrosis. The mechanisms underlying KGF's protective effect in the lung are not completely understood. KGF has plieotropic effects on alveolar epithelial cells including; mitogenic acitivity, increased surfactant production [31], inhibition of apoptosis [32], and increased transcription and/or translation of the major sodium and chloride transport proteins [33]. However, KGF is not known to have direct anti-inflammatory activity. We postulate that TSG-6 and KGF secreted by hMSCs cooperate to attenuate lung injury by suppressing inflammation and promoting lung repair respectively. MSCs secrete other bioactive molecules such as IL1RN (Figure 5c), HGF, EGF, TGF-β_1_, sTNFR1, Ang1 and STC-1 that may also contribute to their immunomodulatory functions and repair of injured lung [19,26,34-38]. Additional experiments are required to determine the relative contribution of each of these factors to the beneficial effects of hMSCs in the lung.

It is tempting to speculate that strategies to enhance hMSC expression of TSG-6 and other beneficial factors such as KGF may improve their lung protective properties. One such strategy is to culture hMSCs as 3D aggregates or as spheroids [39-41]. Recently it was shown that hMSCs cultured in hanging drops formed spheroids and expressed significantly higher levels of TSG-6 and STC-1 compared to hMSCs cultured as monolayers. Moreover, hMSCs from dissociated spheroids were more effective at suppressing inflammation in a mouse model of peritonitis than were hMSCs cultured as adherent monolayers [42]. Using a similar strategy we showed that culture of hMSCs in spheroids increased their expression of TSG-6, STC-1 and KGF (unpublished data). Our finding that hMSCs delivered IP have beneficial effects in the lung suggests the possibility of transplantation of the intact spheroids. We are currently comparing the therapeutic potential of traditionally cultured hMSCs to that of spheroid hMSCs in our animal model of ALI.

## Conclusions

This study demonstrates that xenographic transplantation of human MSCs, by multiple routes, attenuates inflammation and lung injury in an *in vivo *model of acute lung injury. Identification of TSG-6 as a major mediator of the beneficial effects of hMSCs in the lung furthers our understanding of how hMSCs work and supports the concept that the protective effects of MSCs are largely mediated through production of paracrine factors. It is unlikely that a single factor mediates the beneficial effects of hMSCs. MSCs respond to their environment by secreting a variety of factors referred to as the secretome. Elucidation of the hMSC secretome will likely provide strategies to improve the therapeutic potential of hMSCs for treatment of lung injury.

In conclusion, our study provides important preclinical data for potential clinical trials using allogeneic hMSCs for treatment of ALI, a significant source of morbidity and mortality in critically ill patients.

## Abbreviations

ALI: acute lung injury; ARDS: acute respiratory distress syndrome; BAL: bronchoalveolar lavage; HLFs: human lung fibroblasts; IL-1RN: interleukin 1 receptor antagonist; KGF: keratinocyte growth factor; LPS: lipopolysaccharide; MPO: myeloperoxidase; MSCs: multipotent stromal cells; OA: oropharyngeal aspiration; *TNAIP6*/TSG-6: TNF-α-induced protein 6; TNF-α: tumor necrosis factor-α.

## Competing interests

DJP is a co-founder of Temple Therapeutics LLC and has pending provisional patent applications filed for therapeutic uses of MSCs and TSG-6. The other authors declare no competing interests.

## Authors' contributions

SD, FH, RS and AR performed experiments and collected data. JHY carried out the microarray analysis. RWB carried out the threshold image analysis of lung injury. SD and DES drafted the manuscript. JAL, BAB, DAW, DJP and DES contributed to conception and design of the study, interpretation of data and editing of the manuscript. All authors approved the final manuscript.

## Supplementary Material

Additional file 1 **Supplemental Figure S1**. Threshold image analysis of LPS-induced lung injury treated with hMSCs. Damage fraction was determined as the ratio of damaged area relative to total parenchyma area. Injured areas determined by microscopic analysis appeared as darkly staining areas macroscopically. Threshold analysis was performed to measure total parenchyma area and injured area. A lung injury index was determined as the percent damaged area relative to total parenchyma area. (**a, b, c**) RGB images of whole H&E-stained sections. Large blood vessels, airway elements, and connective structures have been cleared and appear as background. (**d, e, f**) Threshold digital highlighting of damaged areas. (**g**) Microscopic view of damaged area. The scale bar represents 50 μm. The inset shows the whole section, and the red box marks an area of lung injury and the blue box marks an area with undetectable injury. Click here for file

Additional file 2 **Supplemental Table S1**. Levels of mouse cytokine/chemokine (pg/ml) in the BAL fluid of LPS-exposed lungs (24 h after exposure) treated with hMSCs or PBS. Click here for file

Additional file 3 **Supplemental Figure S2**. IL-6 and MCP-1 ELISA analysis. BAL fluid from mice treated with PBS + PBS, LPS + PBS, LPS + HLFs, LSP + hMSCs (same animals as in Figure 2) was analyzed by individual ELISAs specific for (**a**) murine IL-6 or (**b**) murine MCP-1. The levels of cytokines measured by the multiplex immunoassay and the ELISAs were very similar. HLFs had no effect on the levels of either cytokine. Data are expressed as mean ± SEM (n *= *6 per group). *P < 0.05 compared with LPS + PBS treated mice; † *P *< 0.05 compared with LPS + HLF treated mice, and ‡ *P *< 0.01 versus mice treated with PBS + PBS. Click here for file

Additional File 4 **Supplemental Figure S3**. Heat map of human microarray assays of mouse lungs. RNA was isolated from whole lungs of LPS- (LPS + hMSC) or PBS-exposed (PBS + hMSC) mice 12 h after OA administration of 5 × 10^5 ^hMSCs. RNA samples (approximately 3 μg of total RNA) were assayed on a human (HG-U133 Plus 2.0) microarray (Affymetrix, Santa Clara, CA, USA). An aliquot of total RNA extracted from LPS-exposed lung treated with PBS was also included to detect cross-hybridization of mouse RNA to the human chip. Click here for file

Additional File 5 **Supplemental Figure S4**. Efficient knockdown of human TSG-6 mRNA by RNA interference. hMSCs were transfected with TSG-6 siRNA (hMSCs/TSG-6 siRNA), control non-silencing siRNA (hMSCs/NS siRNA), or mock transfected (hMSCs/No siRNA). Twenty-four hours after transfection human TSG-6 mRNA was analyzed by qRT-PCR. Data are presented as means ± SEM (n = 3). † *P *< 0.05 compared with mice treated with hMSCs/No siRNA, and ‡ *P *< 0.05 versus mice treated with hMSCs/NS siRNA. Click here for file

Additional File 6 **Supplemental Table S2**. Levels of mouse cytokine/chemokine (pg/ml) in the BAL fluid of LPS-exposed lungs (24 h after exposure) treated with hMSCs transfected with TSG-6 siRNA (hMSCs/TSG-6 siRNA), transfected with control non-silencing siRNA (hMSCs/NS siRNA), or mock transfected (hMSCs/No siRNA). Click here for file
