# Supplementary figures and images for: Human multipotent stromal cells attenuate lipopolysaccharide-induced acute lung injury in mice via secretion of tumor necrosis factor-α-induced protein 6
Source: Stem Cell Res Ther. 2011 May 13;2(3):27. doi: 10.1186/scrt68 (PMC3218818; doi:10.1186/scrt68)

# Supplemental Figure 1

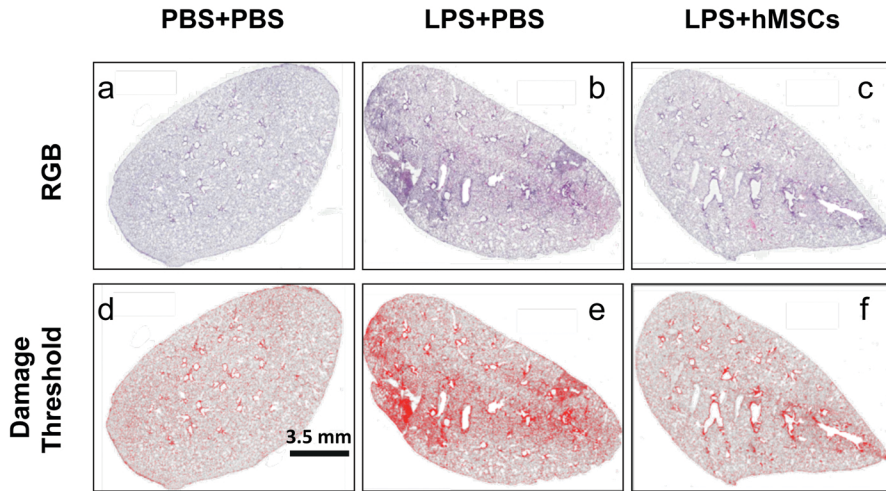

g

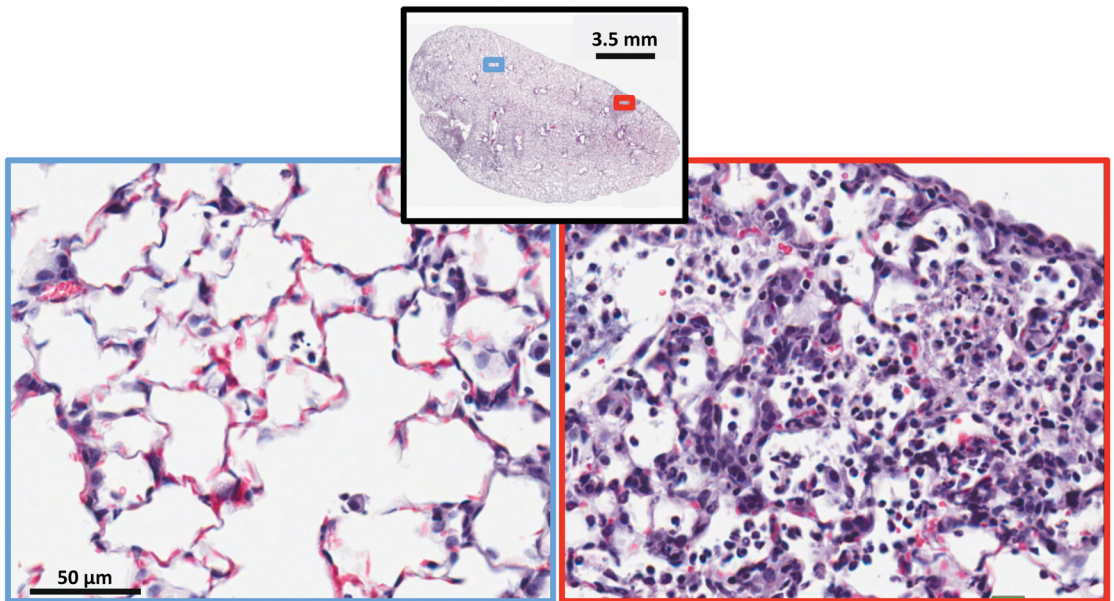

Supplement: Additional file 1 — Supplemental Figure S1. Threshold image analysis of LPS-induced lung injury treated with hMSCs. Damage fraction was determined as the ratio of damaged area relative to total parenchyma area. Injured areas determined by microscopic analysis appeared as darkly staining areas macroscopically. Threshold analysis was performed to measure total parenchyma area and injured area. A lung injury index was determined as the percent damaged area relative to total parenchyma area. (a, b, c) RGB images of whole H&E-stained sections. Large blood vessels, airway elements, and connective structures have been cleared and appear as background. (d, e, f) Threshold digital highlighting of damaged areas. (g) Microscopic view of damaged area. The scale bar represents 50 μm. The inset shows the whole section, and the red box marks an area of lung injury and the blue box marks an area with undetectable injury. [file scrt68-S1.PDF]

*Supplemental Figure 2*

**a**

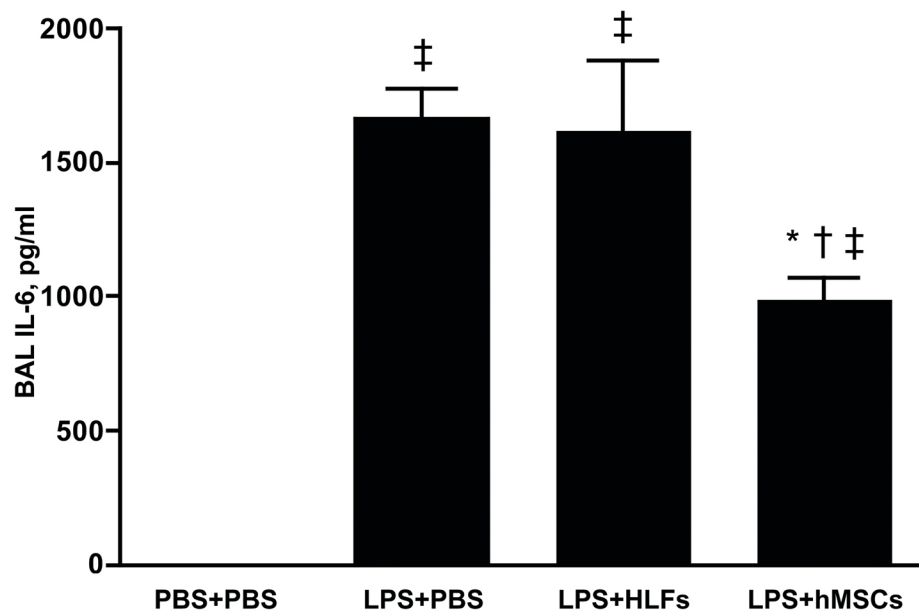

**b**

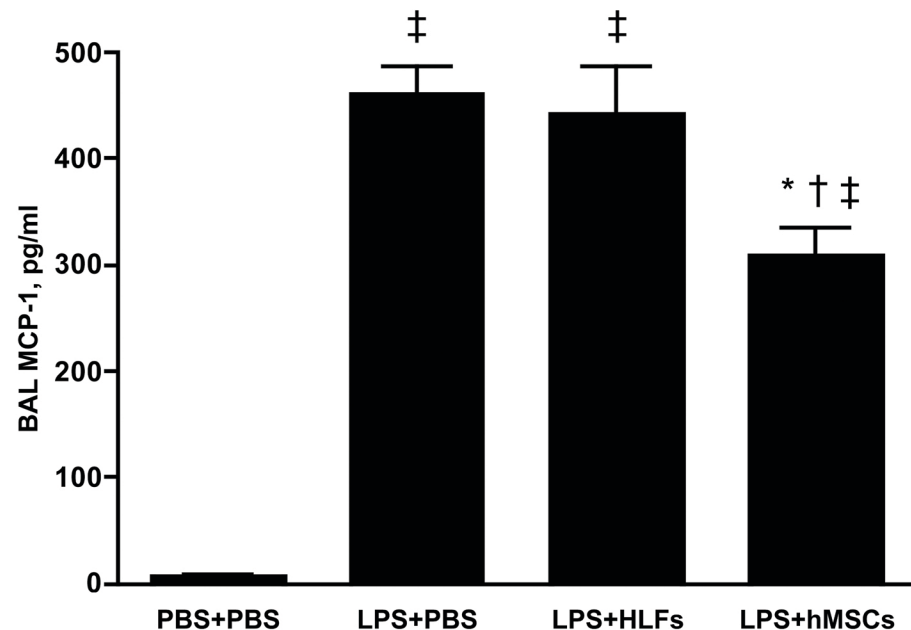

Supplement: Additional file 3 — Supplemental Figure S2. IL-6 and MCP-1 ELISA analysis. BAL fluid from mice treated with PBS + PBS, LPS + PBS, LPS + HLFs, LSP + hMSCs (same animals as in Figure 2) was analyzed by individual ELISAs specific for (a) murine IL-6 or (b) murine MCP-1. The levels of cytokines measured by the multiplex immunoassay and the ELISAs were very similar. HLFs had no effect on the levels of either cytokine. Data are expressed as mean ± SEM (n = 6 per group). *P < 0.05 compared with LPS + PBS treated mice; † P < 0.05 compared with LPS + HLF treated mice, and ‡ P < 0.01 versus mice treated with PBS + PBS. [file scrt68-S3.PDF]

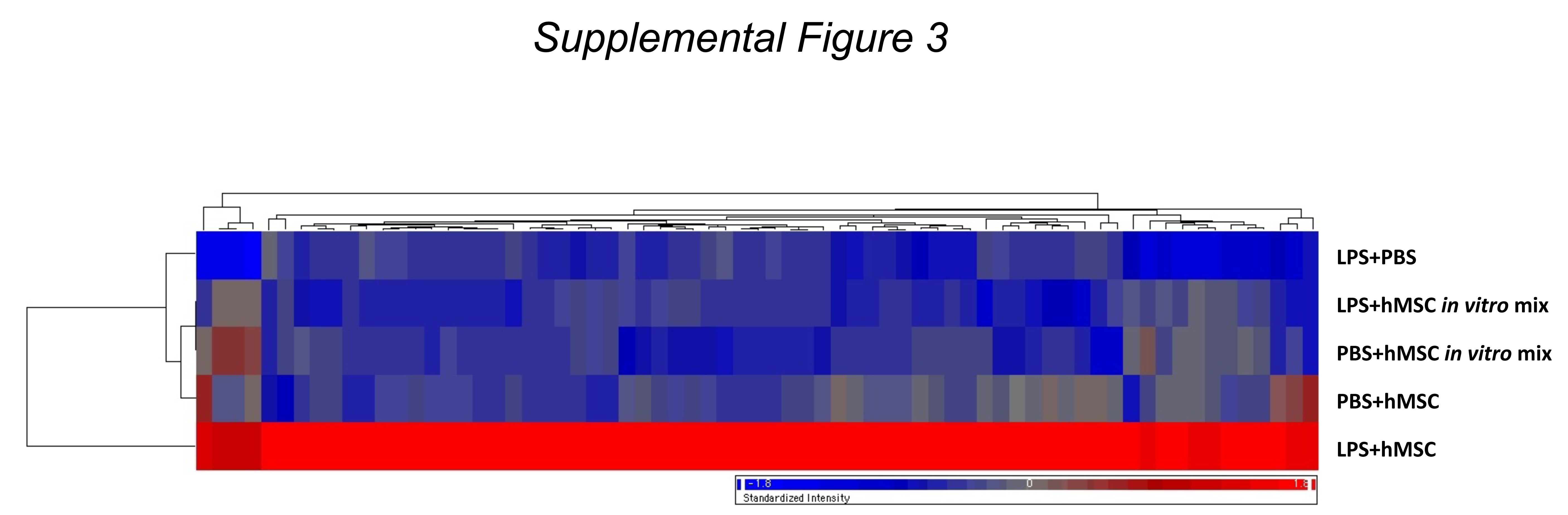

Supplement: Additional File 4 — Supplemental Figure S3. Heat map of human microarray assays of mouse lungs. RNA was isolated from whole lungs of LPS- (LPS + hMSC) or PBS-exposed (PBS + hMSC) mice 12 h after OA administration of 5 × 10^5^ hMSCs. RNA samples (approximately 3 μg of total RNA) were assayed on a human (HG-U133 Plus 2.0) microarray (Affymetrix, Santa Clara, CA, USA). An aliquot of total RNA extracted from LPS-exposed lung treated with PBS was also included to detect cross-hybridization of mouse RNA to the human chip. [file scrt68-S4.TIFF]

## *Supplemental Figure 4*

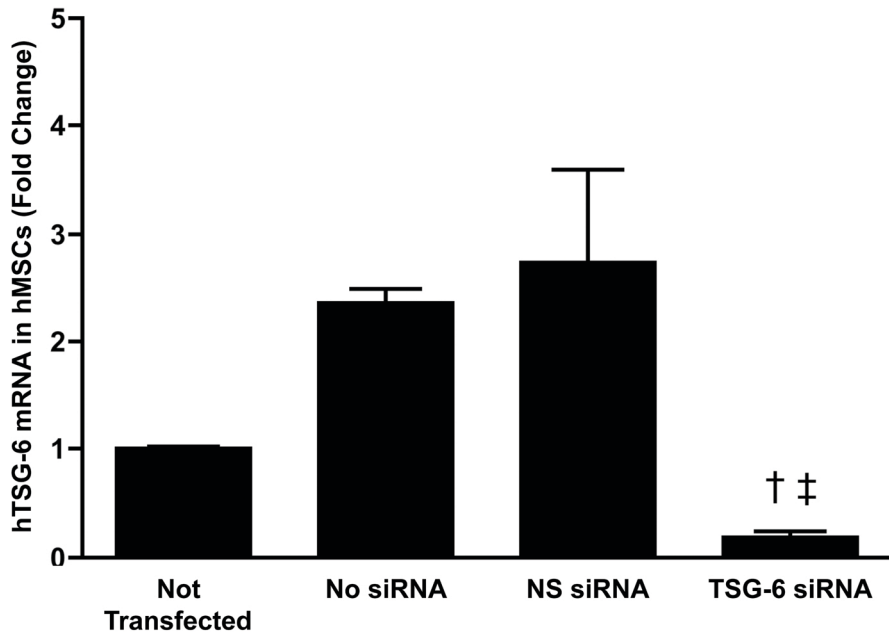

Supplement: Additional File 5 — Supplemental Figure S4. Efficient knockdown of human TSG-6 mRNA by RNA interference. hMSCs were transfected with TSG-6 siRNA (hMSCs/TSG-6 siRNA), control non-silencing siRNA (hMSCs/NS siRNA), or mock transfected (hMSCs/No siRNA). Twenty-four hours after transfection human TSG-6 mRNA was analyzed by qRT-PCR. Data are presented as means ± SEM (n = 3). † P < 0.05 compared with mice treated with hMSCs/No siRNA, and ‡ P < 0.05 versus mice treated with hMSCs/NS siRNA. [file scrt68-S5.PDF]
