# Supplementary material for: Human multipotent stromal cells attenuate lipopolysaccharide-induced acute lung injury in mice via secretion of tumor necrosis factor-α-induced protein 6
Source: Stem Cell Res Ther. 2011 May 13;2(3):27. doi: 10.1186/scrt68 (PMC3218818; doi:10.1186/scrt68)
Supplement: Additional file 2 — Supplemental Table S1. Levels of mouse cytokine/chemokine (pg/ml) in the BAL fluid of LPS-exposed lungs (24 h after exposure) treated with hMSCs or PBS. [file scrt68-S2.DOCX]

| **CYTOKINE/**  **CHEMOKINE** | **TREATMENT** | | | | | |
| --- | --- | --- | --- | --- | --- | --- |
|  | **LPS+PBS** | | | **LPS+hMSCs** | | |
| **G-CSF** | 3750.22 | ± | 370.99 | 2777.79 | ± | 245.72* |
| **GM-CSF** | 71.55 | ± | 10.36 | 88.43 | ± | 13.26 |
| **INF-γ** | ND |  |  | ND |  |  |
| **IL-1α** | 501.52 | ± | 43.39 | 382.46 | ± | 30.47* |
| **IL-1β** | 140.30 | ± | 32.32 | 55.67 | ± | 12.54* |
| **IL-2** | 13.19 | ± | 1.22 | 10.74 | ± | 0.79 |
| **IL-4** | 0.69 | ± | 0.11 | 0.59 | ± | 0.06 |
| **IL-5** | 6.62 | ± | 0.74 | 6.31 | ± | 0.94 |
| **IL-6** | 1951.16 | ± | 223.59 | 1190.50 | ± | 109.06* |
| **IL-7** | 16.10 | ± | 2.17 | 10.14 | ± | 1.02* |
| **IL-9** | ND |  |  | ND |  |  |
| **IL-10** | 7.40 | ± | 1.02 | 8.13 | ± | 1.62 |
| **IL-12 (p70)** | 30.35 | ± | 2.76 | 21.01 | ± | 1.27* |
| **IL-13** | 66.90 | ± | 5.57 | 46.03 | ± | 3.08* |
| **IL-15** | ND |  |  | ND |  |  |
| **IL-17** | 30.78 | ± | 3.11 | 19.05 | ± | 3.14* |
| **IP-10** | 912.80 | ± | 128.45 | 371.56 | ± | 23.18* |
| **KC** | 2332.21 | ± | 138.14 | 2192.09 | ± | 96.21 |
| **MCP-1** | 315.59 | ± | 15.12 | 242.23 | ± | 11.54* |
| **MIP-1α** | 824.77 | ± | 32.04 | 617.99 | ± | 37.96* |
| **RANTES** | 76.74 | ± | 4.24 | 63.26 | ± | 2.63* |
| **TNF- α** | 497.03 | ± | 140.93 | 474.32 | ± | 104.57 |

**Supplemental Table 1.** Levels of mouse cytokine/chemokine (pg/ml) in the BAL fluid of LPS-exposed lungs (24h after exposure) treated with hMSCs or PBS.

BAL, bronchoalveolar lavage; LPS, lipopolysaccharide; PBS, phosphate buffered saline; hMSCs, human multipotent stromal cells; ND, non-detectable; *, p<0.05 vs. LPS+PBS
