# Supplementary material for: Human multipotent stromal cells attenuate lipopolysaccharide-induced acute lung injury in mice via secretion of tumor necrosis factor-α-induced protein 6
Source: Stem Cell Res Ther. 2011 May 13;2(3):27. doi: 10.1186/scrt68 (PMC3218818; doi:10.1186/scrt68)
Supplement: Additional File 6 — Supplemental Table S2. Levels of mouse cytokine/chemokine (pg/ml) in the BAL fluid of LPS-exposed lungs (24 h after exposure) treated with hMSCs transfected with TSG-6 siRNA (hMSCs/TSG-6 siRNA), transfected with control non-silencing siRNA (hMSCs/NS siRNA), or mock transfected (hMSCs/No siRNA). [file scrt68-S6.DOC]

**Supplemental Table 2**. Levels of mouse cytokine/chemokine (pg/ml) in the BAL fluid of LPS-exposed lungs (24h after exposure) treated with hMSCs transfected with TSG-6 siRNA (hMSCs/TSG-6 siRNA), transfected with control non-silencing siRNA (hMSCs/NS siRNA), or mock transfected (hMSCs/No siRNA).

| **CYTOKINE/**  **CHEMOKINE** | **TREATMENT** | | | | | | | | | | | |
| --- | --- | --- | --- | --- | --- | --- | --- | --- | --- | --- | --- | --- |
|  | **LPS+PBS** | | | **LPS+hMSCs/**  **No siRNA** | | | **LPS+hMSCs/**  **NS siRNA** | | | **LPS+hMSCs/**  **TSG-6 siRNA** | | |
| **G-CSF** | 7081.05 | ± | 641.95 | 4048.01 | ± | 496.87* | 4175.5 | ± | 423.79* | 5544.75 | ± | 457.06 |
| **GM-CSF** | 100.17 | ± | 9.31 | 51.99 | ± | 6.93* | 93.64 | ± | 13.46 | 88.44 | ± | 6.14 |
| **INF-γ** | ND |  |  | ND |  |  | ND |  |  | ND |  |  |
| **IL-1α** | 695.11 | ± | 58.53 | 624.67 | ± | 43.16 | 501.04 | ± | 55.87* | 514.74 | ± | 26.89* |
| **IL-1β** | 23.75 | ± | 2.23 | 15.97 | ± | 1.95* | 16.70 | ± | 2.47 | 14.63 | ± | 2.50* |
| **IL-2** | 11.28 | ± | 2.9 | 8.35 | ± | 1.00 | 7.17 | ± | 1.24 | 7.73 | ± | 0.41 |
| **IL-4** | 0.54 | ± | 0.05 | 0.45 | ± | 0.10 | 0.41 | ± | 0.05 | 0.33 | ± | 0.06* |
| **IL-5** | 11.83 | ± | 3.08 | 7.95 | ± | 1.52 | 7.25 | ± | 1.28 | 8.67 | ± | 1.86 |
| **IL-6** | 811.12 | ± | 102.06 | 1004.63 | ± | 86.56 | 963.23 | ± | 80.57 | 1089.25 | ± | 125.83 |
| **IL-7** | 18.41 | ± | 1.45 | 12.27 | ± | 1.76* | 13.20 | ± | 0.77* | 12.25 | ± | 0.71* |
| **IL-9** | ND |  |  | ND |  |  | ND |  |  | ND |  |  |
| **IL-10** | 4.49 | ± | 0.37 | 6.37 | ± | 1.90 | 2.94 | ± | 1.45 | 4.47 | ± | 1.05 |
| **IL-12(p70)** | 20.38 | ± | 1.47 | 14.81 | ± | 1.45* | 14.83 | ± | 1.21* | 17.59 | ± | 1.24 |
| **IL-13** | 57.26 | ± | 4.94 | 46.57 | ± | 2.49 | 39.88 | ± | 3.79* | 45.29 | ± | 2.87* |
| **IL-15** | ND |  |  | ND |  |  | ND |  |  | ND |  |  |
| **IL-17** | 14.91 | ± | 1.84 | 9.73 | ± | 0.93* | 10.11 | ± | 1.22* | 10.99 | ± | 1.11 |
| **IP-10** | 216.76 | ± | 4.03 | 182.07 | ± | 3.50* | 199.16 | ± | 5.32* | 228.66 | ± | 9.91 |
| **KC** | 2055.79 | ± | 166.73 | 2249.29 | ± | 254.73 | 2343.22 | ± | 154.56 | 1853.75 | ± | 81.15 |
| **MCP-1** | 390.24 | ± | 50.74 | 215.58 | ± | 16.77* | 230.49 | ± | 18.32* | 310.76 | ± | 20.08 |
| **MIP-1α** | 1117.28 | ± | 524.91 | 1277.24 | ± | 212.53 | 963.83 | ± | 97.88 | 937.01 | ± | 139.55 |
| **RANTES** | 104.88 | ± | 5.13 | 73.12 | ± | 4.34* | 75.82 | ± | 4.22* | 95.22 | ± | 5.98 |
| **TNF- α** | 963.09 | ± | 111.01 | 1246.83 | ± | 122.58 | 951.69 | ± | 48.40 | 1160.03 | ± | 86.39 |

BAL, bronchoalveolar lavage; LPS, lipopolysaccharide; PBS, phosphate buffered saline; hMSCs, human multipotent stromal cells; TSG-6. TNF-α-induced protein 6; siRNA, small interfering RNA; ND, non-detectable; *, p<0.05 vs. LPS+PBS
